# Wear Behavior of a Heat-Treatable Al-3.5Cu-1.5Mg-1Si Alloy Manufactured by Selective Laser Melting

**DOI:** 10.3390/ma14227048

**Published:** 2021-11-20

**Authors:** Pei Wang, Yang Lei, Jun-Fang Qi, Si-Jie Yu, Rossitza Setchi, Ming-Wei Wu, Jürgen Eckert, Hai-Chao Li, Sergio Scudino

**Affiliations:** 1 Additive Manufacturing Institute, College of Mechatronics and Control Engineering, Shenzhen University, Shenzhen 518060, China; peiwang@szu.edu.cn (P.W.); leiyang_szu@163.com (Y.L.); qijunfangtx@163.com (J.-F.Q.); yusijie1@email.szu.edu.cn (S.-J.Y.); 2 Institute for Complex Materials, Leibniz IFW Dresden, Helmholtzstraße 20, 01069 Dresden, Germany; s.scudino@ifw-dresden.de; 3 Cardiff School of Engineering, Cardiff University, Cardiff CF24 3AA, UK; Setchi@cardiff.ac.uk; 4 Department of Materials and Mineral Resources Engineering, National Taipei University of Technology, No. 1, Sec. 3, Zhong-Xiao E. Rd., Taipei 10608, Taiwan; 5 Erich Schmid Institute of Materials Science, Austrian Academy of Sciences, Jahnstraße 12, A-8700 Leoben, Austria; 6 Department of Materials Science, Chair of Materials Physics, Montanuniversität Leoben, Jahnstraße 12, A-8700 Leoben, Austria; 7 School of Materials Engineering, Shanghai University of Engineering Science, Shanghai 201620, China; 007lihaichao@163.com

**Keywords:** selective laser melting, Al-Si-Cu-Mg alloy, heat treatment, microstructure, wear behavior

## Abstract

In this study, the wear behavior of a heat-treatable Al-7Si-0.5Mg-0.5Cu alloy fabricated by selective laser melting was investigated systematically. Compared with the commercial homogenized AA2024 alloy, the fine secondary phase of the SLM Al-Cu-Mg-Si alloy leads to a low specific wear rate (1.8 ± 0.11 × 10^−4^ mm^3^(Nm)^−1^) and a low average coefficient of friction (0.40 ± 0.01). After the T6 heat treatment, the SLM Al-Cu-Mg-Si alloy exhibits a lower specific wear rate (1.48 ± 0.02 × 10^−4^ mm^3^(Nm)^−1^), but a similar average coefficient of friction (0.34 ± 0.01) as the heat-treated AA2024 alloy. Altogether, the SLM Al-3.5Cu-1.5Mg-1Si alloy is suitable for the achievement of not only superior mechanical performance, but also improved tribological properties.

## 1. Introduction

Selective laser melting (SLM) is one of the advanced additive manufacturing technologies that works with the assistance of computer-aided designs (CAD) and has been widely used for the fabrication of metallic components [1,2]. In recent years, the development of novel alloys fabricated by SLM has attracted a lot of attention due to the outstanding advantages of process flexibility, cost reduction, and energy-saving [3,4]. In addition, Al-based alloys have become one of the most researched SLM materials [5,6], due to the demand and widespread applications in the aerospace and automotive industries.

The low density, good castability, and weldability of SLM-processed Al-Si alloys, as well as their high wear and corrosion resistance, have been studied extensively [7,8]. Prashanth et al. [9] revealed that SLM processing of Al-12Si results in a fine cellular structure (cell size of 1 ± 0.3 μm) yielding three-times higher strength values than the yield strength (260 MPa) of conventional cast samples. Moreover, the tribological properties of Al-12Si samples produced by SLM were evaluated via sliding and fretting wear tests and weight loss experiments, revealing that the as-prepared SLM samples show better wear resistance than their cast counterparts [10]. Similar to SLM Al-12Si, SLM Al-10Si-Mg can also achieve a higher strength reaching up to 500 MPa and a significantly improved wear resistance due to the finer microstructure of the SLM material [11,12,13]. In fact, almost all Al-Si and Al-Si-Mg series alloys, such as Al-35Si, Al-50Si, and Al-7Si-Mg, have significantly higher mechanical performance and wear resistance compared with the Al-Cu and Al-Zn series alloys [14,15,16]. However, the mechanical properties and wear resistance of the current Al-Si and Al-Si-Mg series alloys need additional effort to be tailored to meet the demands that the Al-based alloys require under working conditions [11,17,18].

In recent years, Al-Cu series aluminum alloys have been researched due to their good heat treatability, excellent specific strength, and good damage tolerance [19,20,21]. However, the elimination of cracks in Al-Cu series aluminum alloys resulting from the high cooling rate of SLM (10^4^–10^5^ K/s [22]) is considered as the most important challenge of Al-Cu series aluminum alloys that are fabricated by SLM. Furthermore, Al-Cu series aluminum alloys as construction materials will inevitably encounter friction and wear conditions [1,23]. Therefore, the design and fabrication of SLM Al-Cu series aluminum alloys with high strength and improved wear resistance has important practical significance.

The addition of Si can decrease the melting temperature of Al alloys, which is helpful in decreasing the thermal stresses during the processing of SLM and in refining their grain size. This can prevent hot cracking in SLM Al-Cu alloys during the processing of SLM [17,24]. Moreover, the addition of Si can lead to the formation of Mg_2_Si phase with high melting temperature, low density, high hardness, low thermal expansion coefficient, and reasonably high elastic modulus [25]. The formation of Mg_2_Si phase and the grain refinement of Al alloys may not only contribute to an optimized combination of strength and ductility of Al alloys, but may also improve their wear resistance [26,27]. Therefore, a SLM Al-3.5Cu-1.5Mg-1Si alloy with improved mechanical properties and corrosion resistance was designed and fabricated in our previous work [28,29].

In the present work, the wear resistance of the Al-3.5Cu-1.5Mg-1Si alloy in both as-prepared SLM and heat-treated conditions is investigated systematically and compared with a commercial AA2024 alloy. The effect of microstructural particularities on the wear mechanism of the SLM Al-3.5Cu-1.5Mg-1Si alloy is discussed comprehensively.

## 2. Experimental Details

### 2.1. Materials and SLM Processing

**Figure 1:**
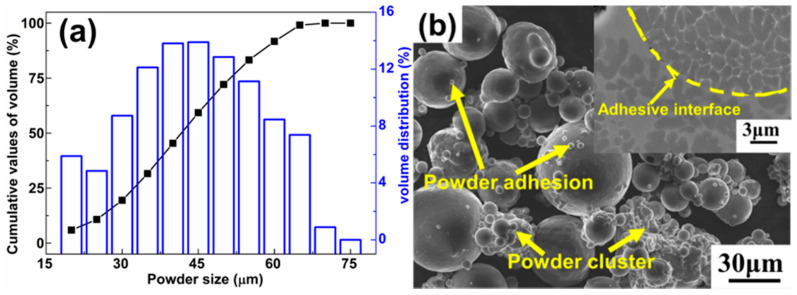
(**a**) Distribution of the powder size; (**b**) typical morphologies of the starting Al-3.5Cu-1.5Mg-1Si powders (inset: Cross-section of a powder particle).

The Al-3.5Cu-1.5Mg-1Si alloy was fabricated using a SLM 250^HL^ device (SLM Solutions Group AG, Luebeck, Germany) equipped with a 400 W Nd:YAG laser (continuous wave, IPG Photonics, Massachusetts, USA), using gas-atomized powders (powder size: 20–60 μm; d_50_ of the powders: 41 ± 1 µm) with a nominal composition of Al-3.5Cu-1.5Mg-1Si (wt%). Figure 1 depicts on the characteristics of the starting gas-atomized Al-3.5Cu-1.5Mg-1Si powder used for SLM processing. More details on the SLM processing of this alloy can be found in [29]. SLM processing was done under protective argon atmosphere with a purity of 99.999%. The oxygen content in the SLM chamber was below 0.02 vol% during the complete processing time. 

As reference material, a homogenized commercial AA2024-O alloy was supplied by Harbin Institute of Technology. Details on its exact composition (4.06 wt% Cu, 1.36 wt% Mg, 0.18 wt% Si, and 0.57 wt% Mn) can be found in [28]. In order to improve the mechanical properties, the as-SLM alloy and AA2024-O alloy samples were solution-treated at 793 K for 1 h, water quenched, and then subjected to aging at 463 K for 10 h, followed by air cooling. These heat-treated samples are termed T6.

### 2.2. Characterization

The analysis of phases and structures was performed by X-ray diffraction (XRD; D3290 PANalytical X’pert PRO, Co-Kα radiation (λ = 0.178897 Å), PANalytical, PANalytical, Almelo, The Netherlands) in reflection mode. In order to maintain a uniform sample height, the bulk samples were grinded to 0.8 ± 0.1 mm. The microstructures were characterized by a high-resolution scanning electron microscope (SEM; Gemini LEO 1530 microscope, Carl Zeiss AG, Oberkochen, Germany). The SEM was equipped with an energy-dispersive X-ray spectroscopy system (EDS; Quantax400 with SDD-Detector Xflash4010, Bruker QUANTAX, Ettlingen, Germany) for element analysis. Based on the imaging principles of SEM, both back-scattered electron (BSE) and secondary electron (SE) modes were used to observe the microstructure.

### 2.3. Mechanical Properties

Vickers microhardness tests were performed using an HMV Shimadzu microhardness tester (Shimadzu Corporation, Tokyo, Japan) with 50 g of load and 10 s of dwell time. The hardness of each sample is the value averaged over at least 20 different points, which were selected automatically. Room-temperature compression tests were carried out with the loading axis parallel to the building direction (BD), using an Instron 5869 machine (Instron, Boston, MA, USA) at a constant crosshead speed of 0.001 mm/s according to the DIN 50106 standard. A laser extensometer (Fiedler Optoelektronik GmbH, Luetzen, Germany) was used to measure the strain directly on the samples. At least five samples were tested in the same condition to ensure the reproducibility of the results.

### 2.4. Sliding Wear Tests

**Figure 2:**
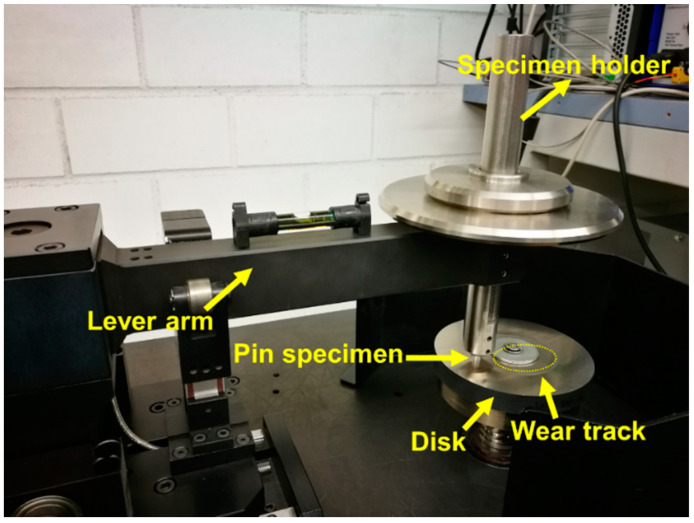
Photograph of the pin-on-disk wear test setup.

Sliding wear tests were carried out by a pin-on-disk setup with a Tribometer T500 (Nanovea, Irvine, CA, USA) at room temperature, according to DIN EN 1071-13 [30,31]. The temperature was detected to control the temperature during the measurement, which was stabilized in the range of 313–323 K. Pin samples made from the tested alloys (Ø6 × 30 mm) and a X210Cr12 steel grinding disk (hardness of 57–62 HRC and elasticity modulus of 210~218 GPa) were used for the measurements, which were performed with the setup shown in Figure 2. Before the wear tests, the samples were grinded by the SiC grinding paper with a final finish of 1200 grit (P4000).

According to DIN EN 1071–13, the specific wear rate *k* was calculated as follows:

(1) $$k=\frac{V}{F_{N}L}$$

A normal load *F_N_* of 15 N was applied on the lever arm during the measurement. The sliding distance is given by *L* = 2*πrvt*, where *r* is the radius of the wear track (30 mm), *v* is the rotating speed of the disk (400 RPM), and *t* is the sliding time (60 min). According to the calculation, the sliding distance (*L*) is 4521 m. Therefore, the wear volume *V* was determined for every tested sample by *V* = (*m_0_ − m*)/*ρ*, whereas *m_0_* and *ρ* are the mass of the pin samples before the wear test and their density, and *m* is the mass after testing. 

In addition, the average value of the coefficient of friction (COF) was measured using a separate load system in contact with the continuous lever arm and recorded by a Nanovea Tribometer system. All of the data (specific wear rate and average COF) shown are averages of at least three different measurements.

## 3. Results and Discussion

**Figure 3:**
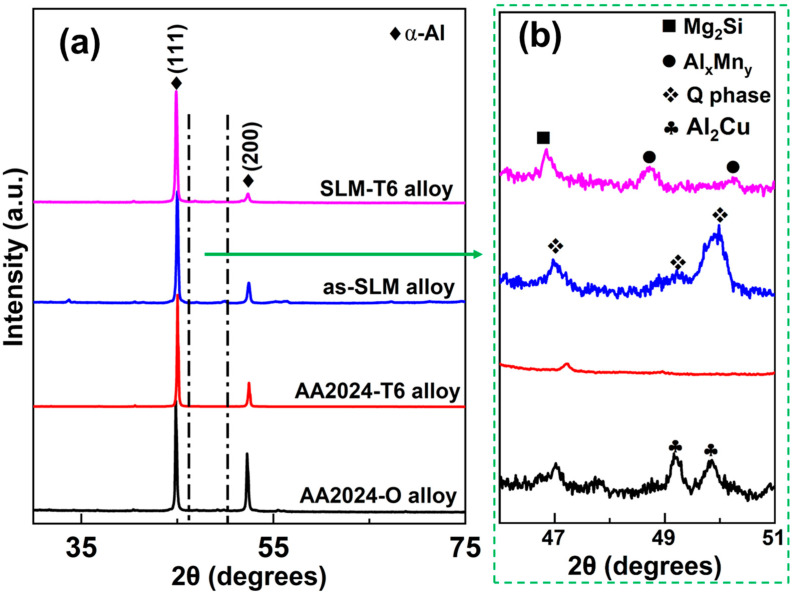
XRD patterns of the SLM Al-3.5Cu-1.5Mg-1Si alloy and of the commercial AA2024-O alloy with and without the T6 heat treatment: (**a**) Overview; and (**b**) close-up view of the diffraction patterns between 46° ≤ 2θ ≤ 51°.

The phase analysis of all the materials is shown in Figure 3, which contains the α-Al phase and Q phase in the XRD patterns of the as-SLM samples [29,32]. After the T6 heat treatment, the SLM-T6 alloy shows the following phases: α-Al, Mg_2_Si (Fm-3m [32,33]), and Al_x_Mn_y_ (unknown space group), suggesting that the Q phase transforms to Mg_2_Si and Al_x_Mn_y_. The details of the phase transformation were discussed in [29]. As shown in Figure 3, there are α-Al and Al_2_Cu phases in the AA2024-O alloy and the Al_2_Cu phases disappear after the T6 treatment. Moreover, the phase with the peak at 47° cannot be identified unambiguously by the X’pert high scores (version 5.1.0, PANanlytical, Almelo, The Netherlands) software. Furthermore, from References [34,35,36], there is only very limited information on this phase. Therefore, the phases with the peak at 47° in AA2024-O and AA2024-T6 were neglected in the XRD patterns.

**Figure 4:**
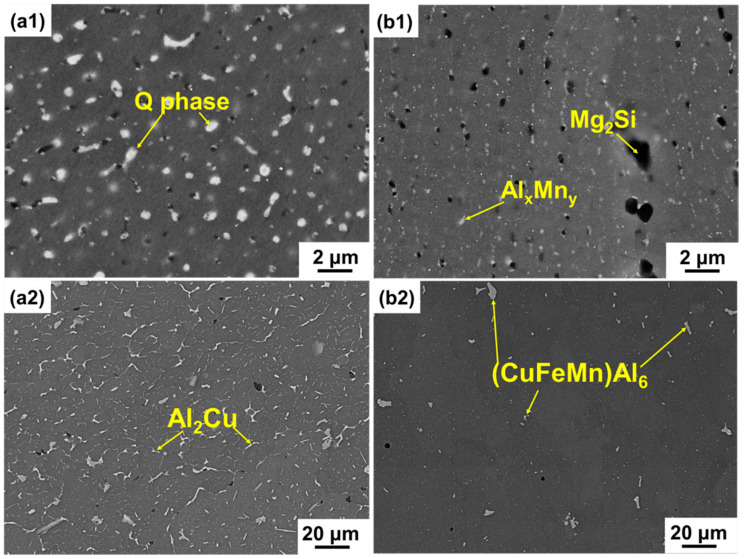
Typical morphologies of the alloys in BSE mode: (**a1**) The as-SLM alloy; (**b1**) SLM-T6 alloy; (**a2**) AA2024-O alloy; (**b2**) AA2024-T6 alloy.

Figure 4 shows the typical microstructures of the SLM Al-Cu-Mg-Si alloy and the AA2024-O alloy before and after the T6 heat treatment. The nano-sized Q phase (around 300 nm), which contains Al, Cu, Mg, and Si elements, is generated in the Al matrix of the as-SLM alloy, due to the rapid solidification during the processing of SLM [29,37]. After the T6 heat treatment, the Al matrix shows the formation of the Al_2_Cu(Mg) (θ’ and S’) phase in the SLM-T6 alloy, which leads to a significant improvement of the tensile strength [29]. In addition, the heat treatment results in transformation of the fine Q phase into Mg_2_Si and Al_x_Mn_y_ (Figure 4b1). It should be mentioned that the formation of these phases, especially of the Mg_2_Si phase, has a significant importance in improving the friction and wear properties of Al-Cu-Mg-Si alloys [38]. Compared with the as-SLM alloy, the white θ-Al_2_Cu phase (EDS measurement: 74.7 ± 1.2 at% Al and 24 ± 1.1 at% Cu) is the main phase dispersed in the AA2024-O alloy (Figure 4a2). After the T6 heat treatment, the Al_2_Cu phase is dissolved in the Al matrix, and undefined (CuFeMn)Al6 phases (EDS measurement containing mainly additional 65.5 ± 0.4 at% Al, 6.2 ± 0.5 at% Cu, 7.4 ± 0.4 at% Mn, and 12.9 ± 0.4 at% Fe) are generated. Similar microstructures and phases were described in previous works on the AA2024 alloy [34,35,36,39,40].

**Figure 5:**
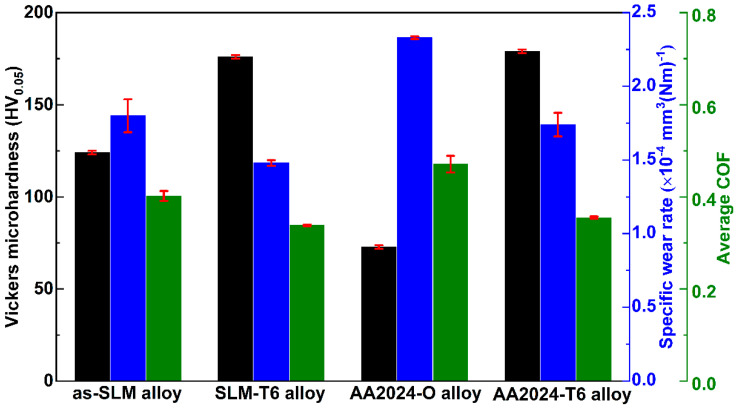
Microhardness, average coefficient of friction (average COF), and specific wear rate of all the samples.

The microhardness, the average coefficient of friction (average COF), and the specific wear rate of all the alloys are shown in Figure 5. The microhardness, the average COF, and the specific wear rate of the AA2024-O alloy and the AA2024-T6 alloy are 73 ± 1 HV_0.05_, 0.47 ± 0.02, 2.23 ± 0.01 × 10^−4^ mm^3^(Nm)^−1^ and 179 ± 1 HV_0.05_, 0.36 ± 0.01, 1.74 ± 0.08 × 10^−4^ mm^3^(Nm)^−1^, respectively. Evidently, the as-SLM alloy has a higher hardness (124 ± 1 HV_0.05_), lower average COF (0.40 ± 0.01), and lower specific wear rate (1.8 ± 0.11 × 10^−4^ mm^3^(Nm)^−1^) than the AA2024-O alloy. After the T6 heat treatment, with the increasing hardness of the SLM-T6 alloy to (176 ± 1 HV_0.05_), the average COF and the specific wear rate decrease to 0.34 ± 0.01 and 1.48 ± 0.02 × 10^−4^ mm^3^(Nm)^−1^, respectively. The formation of nano-Al_2_Cu (Mg) precipitates in the AA2024-T6 alloy and the SLM-T6 alloy enhances the mechanical properties. According to the classical Archard’s equation, the coefficient of friction and the wear loss of a material is inversely proportional to its hardness under adhesive wear conditions [41,42,43]. Therefore, after the heat treatment, the strengthened Al matrix of the SLM-T6 alloy results in an improvement of its frictional and wear properties. It is striking that the AA2024-T6 alloy has a slightly higher hardness, but a relatively lower specific wear rate than the SLM-T6 alloy. In general, the higher Cu and Mg contents (Cu: 4.06 ± 0.01 wt%, Mg: 1.36 ± 0.01 wt% [28]) in the AA2024-T6 alloy can result in more nano-Al_2_Cu (Mg) precipitates in the Al-matrix than the SLM-T6 alloy, contributing to the higher hardness of the AA2024-T6 alloy [44,45]. However, due to the positive effect of the solid solution of Si on the wear properties of the Al-Cu-Mg alloy [38,46,47], the high Si content in the SLM-T6 alloy, especially the formation of Mg_2_Si (Figure 4a2), can lead to a lower average COF and specific wear rate than the AA2024-T6 alloy.

In addition, after the heat treatment, the Q phase in the as-SLM alloy dissolves into the Al matrix, and is accompanied by the disappearance of the classical layer-by-layer microstructure generated during the processing of SLM. Moreover, the nano-precipitates are dispersed uniformly to strengthen the Al matrix [29]. The disappearance of the layer-by-layer microstructure results in a lower standard error of the average of the COF SLM-T6 alloy and a specific wear rate than the as-SLM alloy.

**Figure 6:**
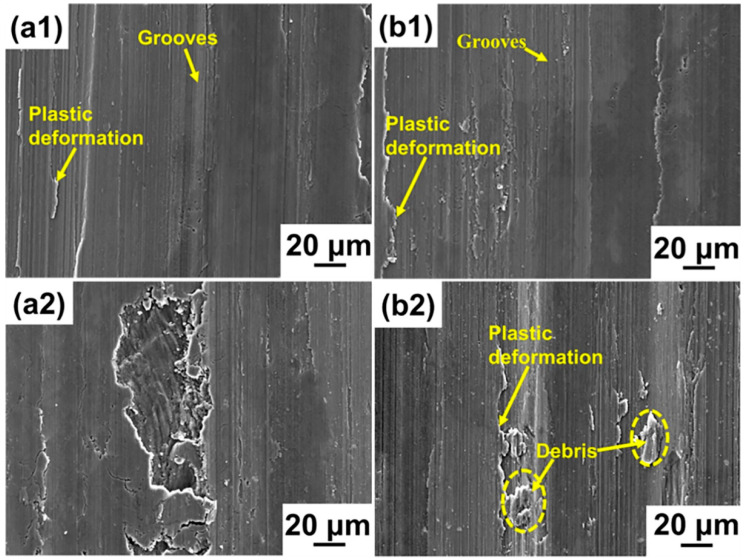
SEM morphologies of the worn surfaces in SE mode: (**a1**) The as-SLM alloy; (**b1**) SLM-T6 alloy; (**a2**) AA2024-O alloy; (**b2**) AA2024-T6 alloy.

In order to compare the wear mechanism of the SLM materials with the commercial alloy, the worn surfaces of all the materials are shown in Figure 6. The grooves in the as-SLM alloy are slim, resulting in a smooth worn surface. After the T6 heat treatment, the wear debris is very fine and the surface is smoother than the as-SLM alloy without the heat treatment. Despite the existence of fine debris for the SLM-T6 alloy, the morphologies of the SLM Al-Cu-Mg-Si specimens with and without the heat treatment mainly show typical abrasive wear features. For comparison, the surface of the AA2024-O alloy not only shows large grooves resulting from plastic deformation, but also a sizeable amount of large debris, representing a classical mixed worn morphology, indicative of abrasive and adhesive mechanisms. Although the formation of the nano-precipitates after the T6 heat treatment improves the hardness of the Al matrix, there is still a sizeable amount of large debris and only an insignificant plastic deformation for the AA2024-T6 alloy. This indicates that despite the improved mechanical properties of the AA2024-T6 alloy, the main wear mechanism of this alloy does not change. The morphological comparison of the SLM-T6 and AA2024-T6 alloys provides evidence for the poor wear properties of the AA2024-T6 alloy despite its higher hardness.

**Figure 7:**
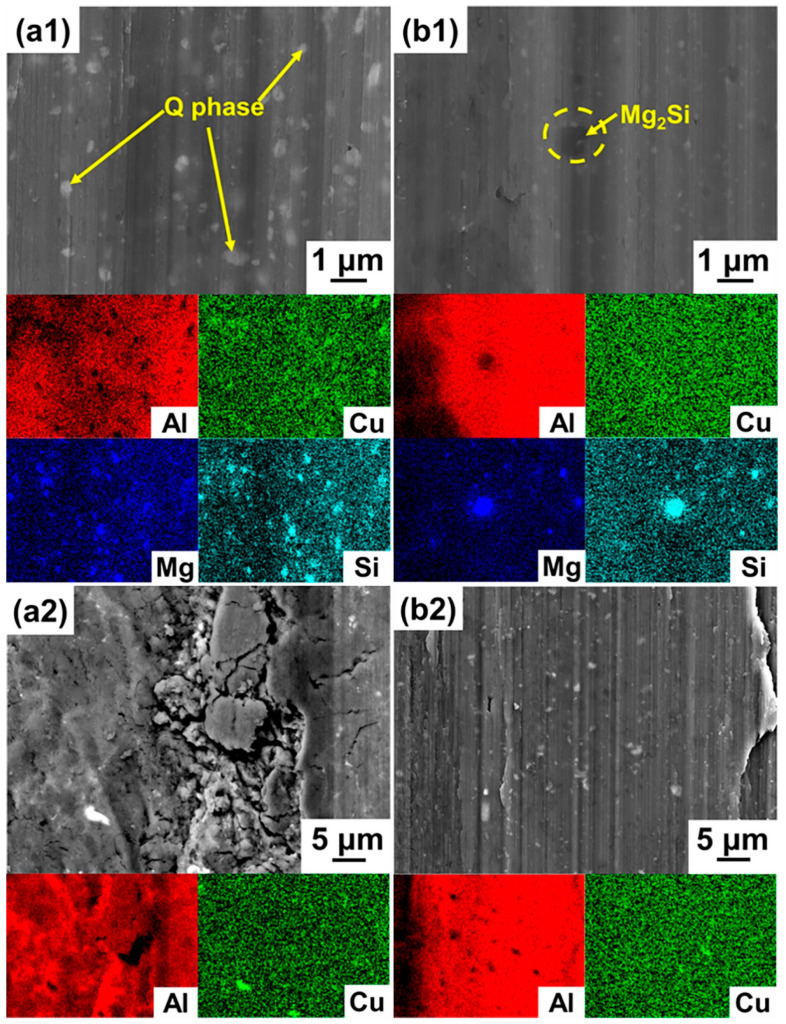
SEM close-up view and EDS maps at high magnification in SE mode: (**a1**) The as-SLM alloy; (**b1**) SLM-T6 alloy; (**a2**) AA2024-O alloy; (**b2**) AA2024-T6 alloy.

In order to analyze the influence of the different phases on the features observed on the worn surfaces and the wear properties, the worn surfaces of the samples at high magnification are shown in Figure 7. The fine Q phase (~200 nm) is embedded into the grooves of the Al matrix after the wear test. After the T6 heat treatment, the grooves only exhibit the area around the Mg_2_Si phase. As shown in (Figure 7b1), the observation of the grooves is difficult around these fine phases, due to the smaller size of the Al_x_Mn_y_ phase. The morphologies at high magnification indicate that the small size of the Q and Mg_2_Si phases, respectively, lead to slim grooves and smooth worn surfaces for the as-SLM and the SLM-T6 alloys. Compared with the as-SLM alloy, the large Al_2_Cu phase (≥2 μm) in the AA2024-O alloy is fragmented during the wear test and the pieces of the Al_2_Cu phase are embedded into the Al matrix. Therefore, due to the synergistic effect of the crushed Al_2_Cu hard phase and the soft Al matrix, the large debris is cut off from the matrix, and subsequently attached on the Al matrix during the wear test (Figure 7a2). Despite the effect of the heat treatment on the strengthening of the Al matrix, the adhesive phenomenon is reduced, but still a few large debris on the worn surface are visible due to the large size of the (CuFeMn)Al_6_ phase (Figure 7b2). According to the above analyses, compared with the commercial AA2024 alloy at the same heat treatment condition, the fine phases can result in the improvement of the wear resistance of the SLM Al-Cu-Mg-Si alloy.

## 4. Conclusions

In this study, the wear resistance of the SLM Al-3.5Cu-1.5Mg-1Si alloy with and without the T6 heat treatment was investigated and their wear mechanisms were discussed. The nano-size Q phase generated during the processing of SLM causes the SLM Al-Cu-Mg-Si alloy to have a better specific wear rate (1.8 ± 0.11 × 10^−4^ mm^3^(Nm)^−1^) and average of coefficient of friction (0.40 ± 0.01) than the conventional AA2024-O alloy at the same heat treatment condition. After the T6 heat treatment, nano-sized Al_2_Cu(Mg) and nano-size Mg_2_Si phases precipitate in the matrix, which lead to the improvement of the wear resistance, i.e., the average COF and the specific wear rate decrease to 0.34 ± 0.01 and 1.48 ± 0.02 × 10^−4^ mm^3^(Nm)^−1^. The morphologies after the wear tests of the SLM Al-3.5Cu-1.5Mg-1Si alloys with and without the T6 heat treatment both show a typical plastic deformation with slim grooves, indicating the occurrence of an abrasive mechanism.

## Data Availability

The raw data required to reproduce these results cannot be shared at this time as the data also forms part of an ongoing study.
